# The IFT20 homolog in *Caenorhabditis elegans* is required for ciliogenesis and cilia-mediated behavior

**DOI:** 10.17912/micropub.biology.000396

**Published:** 2021-05-11

**Authors:** Ana R. G. De-Castro, Joana Quintas-Gonçalves, Tiago Silva-Ribeiro, Diogo R. M. Rodrigues, Maria J. G. De-Castro, Carla M. Abreu, Tiago J. Dantas

**Affiliations:** 1 i3S - Instituto de Investigação e Inovação em Saúde, Universidade do Porto, Porto, Portugal; 2 IBMC - Instituto de Biologia Molecular e Celular, Universidade do Porto, Porto, Portugal

## Abstract

Cilia are microtubule-based organelles that carry out a wide range of critical functions throughout the development of higher animals. Regardless of their type, all cilia rely on a motor-driven, bidirectional transport system known as intraflagellar transport (IFT). Of the many components of the IFT machinery, IFT20 is one of the smallest subunits. Nevertheless, IFT20 has been shown to play critical roles in the assembly of several types of mammalian cilia. Here we show that the IFT20 homolog in *Caenorhabditis elegans*, IFT-20, is also important for correct cilium assembly in sensory neurons. Strikingly, however, we find that IFT-20-deficient animals are able to assemble short, vestigial cilia. In spite of this, we show that practically all IFT-20-deficient animals fail to respond to environmental cues that are normally detected by cilia to modulate their behavior. Altogether, our results indicate that IFT-20 is critical for both the correct assembly and function of cilia in *C. elegans*.

**Figure 1. Disruption of ift-20 results in defective cilium assembly and function in C. elegans:**
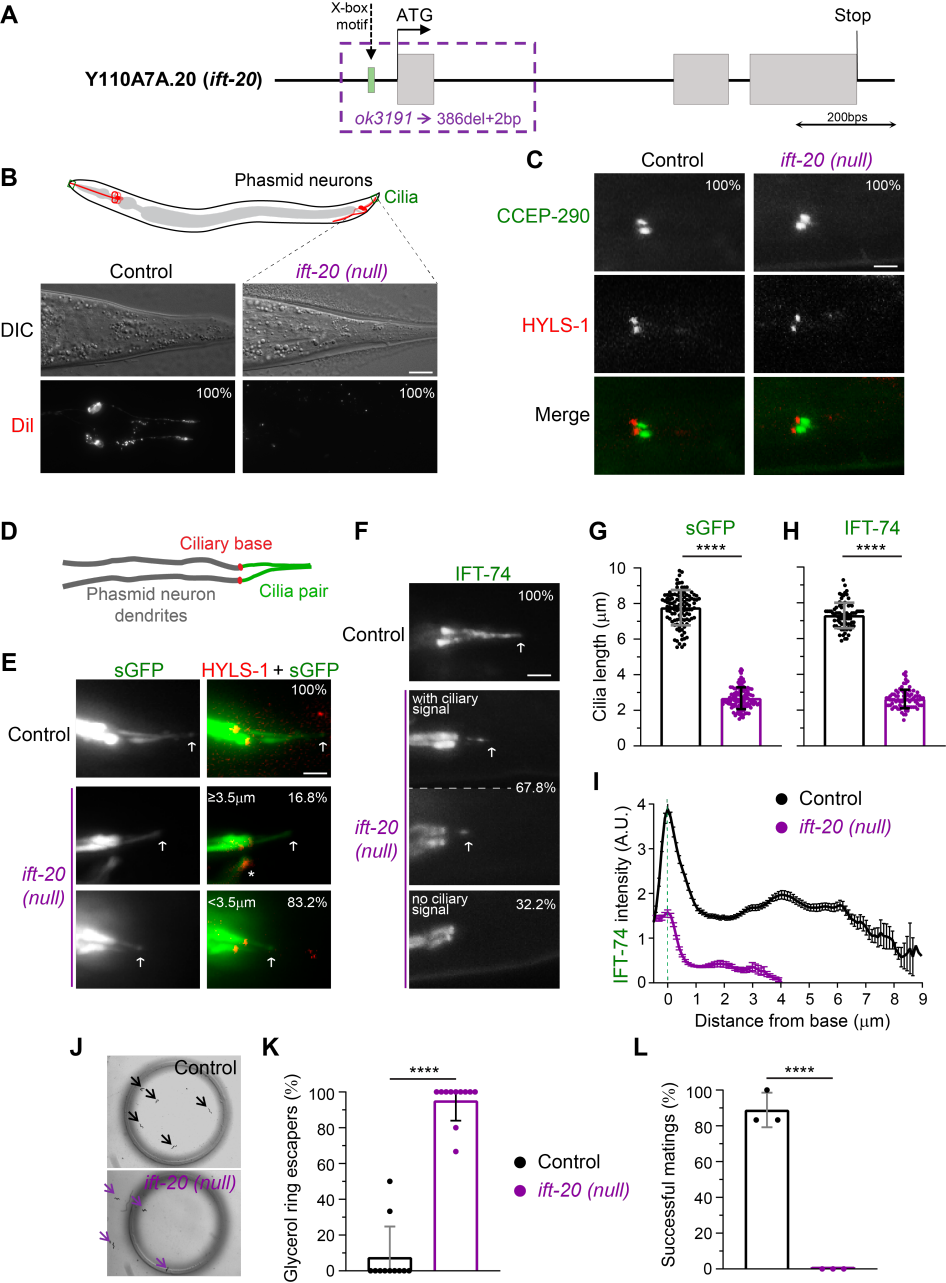
**(A)** Schematic of the Y110A7A.20 locus, showing the predicted *ift-20* exons (in grey boxes) and the relative position of the X-box motif according to (Blacque *et al.* 2005; Efimenko *et al.* 2005). The *ok3191* deletion/substitution allele (-386bp, +2bp=C+T) was mapped and is represented in purple. **(B)** Examples of the DiI lipophilic dye-filling test in sensory phasmid neurons of wild-type and *ift-20* null worms. 100% of the *ift-20* null worms fail to incorporate dye (N≥15 worms per strain). Scale bar, 10 μm. **(C)** Examples of the dendritic ends of phasmid neurons from wild-type and *ift-20* mutants expressing GFP::CCEP-290 and mCherry::HYLS-1, labeling the transition zone and the base of cilia, respectively (N≥18 cilia per strain). Scale bar, 2 μm. **(D)** Illustration of the ciliated dendritic ends of phasmid neurons. **(E)** Examples of cilia of phasmid neurons of wild-type and *ift-20* mutants, labeled with soluble GFP (sGFP; able to enter cilia) and mCherry::HYLS-1 (ciliary base). *refers to a dendrite from a different type of neuron (PQR) also present in the figure. **(F)** Examples of the distribution of endogenously labeled IFT-74::GFP and its incorporation into cilia of phasmid neurons. White arrows point at the tips of cilia.Scale bars, 2 μm. **(G, H)** IFT-20-deficient worms have defects in ciliogenesis but manage to assemble severely shortened cilia as shown in the quantifications (N≥107 sGFP labeled cilia were analyzed per strain; N≥80 cilia with detectable IFT-74::GFP were analyzed per strain). Bars in graphs show the average cilia length (± SD). **(I)** Average IFT-74::GFP signal distribution and intensity (± SEM) along cilia are altered in *ift-20* null worms, revealing that they incorporate less IFT-74 (N≥80 cilia for each strain; worms with no ciliary IFT-74 signalwere not included). **(J)** Osmotic avoidance assay shows that sensory cilia of *ift-20* null worms fail to sense the high glycerol concentrations of the ring surrounding them, and cross it to escape. **(K)** Graph shows the average percentage of worms (± SD) that cannot sense a hypertonic glycerol barrier (N=28 worms in controls; N=35 *ift-20* null worms; analyzed over 11 independent experiments). **(L)** Mating assay was also performed to test the proficiency of sensory cilia functions in *ift-20* null worms. The graph shows the mating success score (± SD) of each experimental repeat: considering 0% (no mating) or 100% (mating) for each hermaphrodite singled-out from a plate containing males of the strain being tested (a total of N=18 male worms from each strain were analyzed, over 3 independent experiments). 100% of the *ift-20* null males fail to mate with wild-type control hermaphrodites. ****P≤0.0001.

## Description

A high diversity of cilium types have evolved to carry out a multitude of sensorial and motility functions in animals. It is therefore not surprising that defects in cilium structure and function have been associated with various severe multi-organ disorders, commonly known as ciliopathies (Sreekumar and Norris 2019). Regardless of their type, cilium assembly relies on bidirectional motor-driven transport known as intraflagellar transport (IFT; Prevo *et al.* 2017). Anterograde IFT driven by kinesin-2 transports different cargos in IFT trains along axonemal microtubules from the ciliary base to the tip of the cilium. Upon reaching the tip, these IFT trains are dismounted, rearranged and prepared for transport in the opposite direction back to the ciliary base by the dynein-2 motor complex. IFT trains are composed of at least 16 IFT-B subunits and 6 IFT-A subunits (Prevo *et al.* 2017). IFT20 is part of the IFT-B complex and has been shown to be of critical importance for ciliogenesis in human and mouse models (Follit *et al.* 2006; Jonassen *et al.* 2008; Katoh *et al.* 2017; Lim *et al.* 2020).

In *C. elegans*, IFT-20 is encoded by the Y110A7A.20 locus on chromosome I. Y110A7A.20 was identified in 2004 as a locus containing a X-box sequence (Fan *et al.* 2004; Blacque *et al.* 2005; Efimenko *et al.* 2005) under the control of DAF-19, a RFX-type transcription factor that regulates ciliary gene expression in ciliated sensory neurons (Swoboda *et al.* 2000) (the only ciliated cells in *C. elegans*). Expression of a GFP reporter under the control of the *ift-20* promoter revealed that IFT-20 expression is restricted to sensory neurons, similarly to other IFT components (Blacque *et al.* 2005). In fact, disruption of *daf-19* resulted in the almost complete loss of expression of this GFP reporter (Blacque *et al.* 2005), confirming that DAF-19 drives IFT-20 expression most likely by recognizing the X-box at position -59 of the *ift-20* promoter (Blacque *et al.* 2005; Efimenko *et al.* 2005). Later, Ou and colleagues constructed a vector for IFT-20::GFP expression, and found that the *C. elegans* IFT20 homolog is also recruited to cilia where it undergoes IFT with similar kinetics to other IFT subunits (Ou *et al.* 2007). However, in contrast to its homolog in mammalian cells, IFT-20 was not detectable at the Golgi and does not seem to interact with the *C. elegans* homolog of GMAP210 (Broekhuis *et al.* 2013), which anchors IFT20 to the Golgi in mammalian cells (Follit *et al.* 2008).

In spite of these early analyses of IFT-20 expression and localization, the extent to which IFT-20 contributes to ciliogenesis in *C. elegans* remains unknown. To dissect the importance of IFT-20, we obtained and mapped a null deletion allele (*ok3191*) of the *ift-20* locus (Figure 1A). This allele carries a deletion of 386 bps that includes part of the 5′ UTR, the X-box motif that controls *ift-20* expression, the start codon, and all of the 1st exon (sequencing data was submitted to WormBase). Because the expression of *ift-20* was previously shown to be under the control of the DAF-19 transcription factor, and since *ok3191* lacks the complete first exon, we expect that this allele results in the complete loss of IFT-20. Thus, we refer to the *ift-20* (*ok3191*) allele as an *ift-20* null.

We started our analysis of *ift-20* null worms by performing the classic dye-filling assay to test whether cilia are able to form normally in the absence of IFT-20. This assay takes advantage of the fact that the lipophilic, fluorescent DiI dye is specifically incorporated into a subset of ciliated sensory neurons through their cilia (if these are present and in contact with the outside environment; Extended Data Figure 1A). In contrast to the wild-type controls that incorporated the dye, none of the *ift-20* null worms had DiI signal in their neurons (Figure 1B; Extended Data Figure 1B). To confirm that this dye-filling phenotype results from ciliary defects rather than abnormal dendritic morphology/extension, we expressed cytoplasmic GFP in sensory neurons (driven by the *osm-6/ift-52* promoter)to analyze their morphology. We found no significant differences in the length and morphology of sensory neurons (Extended Data Figure 1C), which, together with our dye-filling results, suggest that IFT-20 loss leads to defects in sensory cilia.

Using established markers for the cilium base (HYLS-1) and the ciliary transition zone (CCEP-290) (Schouteden *et al.* 2015), we found that *ift-20* null worms still positioned their basal body normally at the extremity of the dendrite in phasmid neurons and were able to form at least the CCEP-290 module of the ciliary transition zone (Figure 1C). We then took advantage of the incorporation of the soluble/cytoplasmic GFP into cilia of phasmid sensory neurons to visualize cilia and measure their length in *ift-20* null worms. Strikingly, we found that severely shortened/vestigial cilia were still able to form in phasmid neurons of *ift-20* null animals (Figure 1D,E,G). This suggests that IFT-20 loss leads to a severe defect in ciliary axoneme assembly but does not completely block ciliogenesis in *C. elegans*.

In order to directly visualize IFT particles in cilia of *ift-20* null animals, we analyzed the distribution of IFT-74::GFP (Yi *et al.* 2017), another subunit of the IFT-B complex. IFT-74::GFP was readily detectable in dendrites of amphid and phasmid neurons, which allowed us to further confirm the normal development and morphology of these neurons in *ift-20* null worms (Extended Data Figure 1D). In controls, IFT-74::GFP was strongly recruited to cilia, labeling the complete axoneme (Figure 1F,H). In contrast, IFT-74::GFP-labeled cilia were severely shortened in the majority of *ift-20* null phasmid neurons (67.8%; Figure 1F,H), consistent with our observations using soluble GFP-labeled cilia. Interestingly, however, no IFT-74::GFP-labeled cilia were detectable in the remaining fraction of phasmid neurons (32.2%; Figure 1F), suggesting that IFT-20 is also required for IFT-74 incorporation into cilia. To further confirm this possibility, we quantified the distribution of any detectable IFT-74::GFP signal in phasmid cilia of *ift-20* null worms. We found that the signal intensity of IFT-74::GFP recruited to the ciliary base and inside cilia of phasmid sensory neurons was indeed strongly reduced in *ift-20* null worms (Figure 1I). These results reveal that, in addition to being important for cilia assembly in *C. elegans*, IFT-20 plays a role in the ciliary recruitment and entry of IFT subunits.

The fact that short cilia could still form in phasmid sensory neurons of *ift-20* null worms was somewhat unexpected given the critical role of IFT20 for the initiation of ciliogenesis in most mammalian models (Follit *et al.* 2006; Jonassen *et al.* 2008; Katoh *et al.* 2017). Interestingly, however, we note that very short flagella with abnormal axonemes (but still motile) can form in sperm of mice with male germline-specific disruption of IFT20 (Zhang *et al.* 2016).

To assess the degree of functionality of the IFT-20-deficient vestigial cilia, we performed osmotic and mating assays.When performing the osmotic avoidance assay, virtually all *ift-20* null worms readily crossed the glycerol rings that wild-type worms avoided (Figure 1J,K). This suggests that IFT-20-deficient cilia fail to sense normally repulsive concentrations of glycerol.In agreement with defective ciliary sensory functions, all *ift-20* null male worms failed to mate (Figure 1L), suggesting that IFT-20-deficient cilia in male tails were unable to detect wild-type hermaphrodites for mating. Together, the results of these behavioral assays strongly suggest that IFT-20 loss completely impairs cilia-dependent functions in *C. elegans*. Although we did not analyze the morphology and IFT of all types of sensory cilia in the *ift-20* null, our analysis of cilia-dependent behavior is consistent with the cilium assembly defects that we observed in *ift-20* null phasmid sensory neurons.

Altogether, our results show that IFT-20 is required for ciliogenesis and cilia sensory functions in *C. elegans*. Our data also suggest that IFT-20 is important for the robust recruitment and entry of IFT subunits into vestigial cilia that assemble in *ift-20* null animals.

## Methods

***Caenorhabditis elegans* maintenance and strain generation**

*C. elegans* strains were maintained on standard nematode growth medium (NGM) plates seeded with *Escherichia coli* OP50 bacteria at 20 °C, and crossed using standard procedures. Mutant genotyping was performed by standard PCR. *C. elegans* strains used in this study are listed in Table 1.

**Fluorescence imaging**

Young adult hermaphrodite worms were immobilized for imaging using 10 mM Levamisol and were placed on a microscope slide with a 5% agarose pad. Imaging of worms and their cilia was carried out over at least 3 independent experiments using an Axio Observer microscope (Zeiss) controlled by ZEN software (Zeiss), equipped with a 63x 1.46 NA objective lens and an Orca Flash 4.0 camera (Hamamatsu). Z-stacks were acquired with 0.4 µm between each z-section for imaging cilia. Z-stack images were processed and analyzed with Fiji software (Image J version 2.0.0-rc-56/1.52 p). Fluorescence signals of cilium-incorporated soluble GFP (with mCherry::HYLS-1), and IFT-74::GFP were used to measure the length of cilia in phasmid neurons (starting at the maximum signal at the base). The IFT-74::GFP signal intensity of each pixel was measured from the base to the tip of each cilium to generate an averaged profile of IFT-74 distribution along cilia.

**Dye-Filling**

Worms from each strain were collected from a confluent but not starved plate and washed in M9 buffer. They were then incubated in 500 µL of DiI (1,1′-Dioctadecyl-3,3,3′,3′-Tetramethylindocarbocyanine Perchlorate) solution at 0.625 µg/ml for 1 hr at room temperature in the dark, with occasional flipping of the tubes. After washing worms in M9 buffer, these were placed on a NGM seeded plate for 1-3 hr at 20 °C, to reduce the background of ingested dye. The neuronal uptake of dye was then examined using the Axio Observer microscope, as described above.

**Mating Assay**

Mating assays were performed as described in (Hodgkin 1983) for quantitative mating efficiency tests. First, 6x young males of the strain to be tested were separated 24 hr prior to the experiment on a 5 cm NGM seeded plate at 20 °C. These males were then placed together with 6x L4 wild-type hermaphrodites on a mating plate (i.e. a 5 cm NGM agar plate with an ~1 cm spot of OP50 seeded at the center of the plate). The worms were then allowed to mate for 24 hr at 20 °C, after which the hermaphrodites were isolated onto a new 5 cm NGM seeded plate and kept at 20 °C for 24 hr to lay eggs. After this period, hermaphrodites were removed and plates with eggs were kept at 25 °C. Two days after, the number of total progeny and the number of male progeny on the plate were counted. In order to obtain enough males to be used in these assays, both strains used for mating contained a *him-8* mutation, which leads to an increase in mis-segregation of the X chromosome during meiosis I (Phillips *et al.* 2005).

**Osmotic Avoidance Assay**

Osmotic avoidance assays were performed on NGM non-seeded plates at 20 °C, following the guidelines of (Sanders *et al.* 2015). Each repeat was carried out using five young adult hermaphrodite worms of each strain isolated before the experiment. Worms were placed inside of a glycerol ring, with a diameter of approximately 1 cm, freshly prepared with a tube dipped in a 59% glycerol solution. Worm behavior was immediately monitored for 5 min using a camera (controlled by the Micro-Manager 1.4 software) to determine whether worms avoided crossing the glycerol ring or not. Worms that left the ring or stayed in contact with its glycerol border for more than 20 sec were classified as escapers. Worms that failed to explore the area within the timeframe of the experiment were excluded.

**Data analysis and statistics**

Statistical analyses of datasets were performed using GraphPad Prism software. The two-tailed student T test was used to analyze the data in the graphs of Figure 1. Differences were considered significant at P values below 5% (*P≤0.05; **P≤0.01; ***P≤0.001; ****P≤0.0001).

## Reagents

**Table 1: List of *C. elegans* strains used in this work.**

**Table:** 

Strain	Genotype	Short description	Source/Ref.
N2	wild-type	ancestral N2 Bristol	CGC
RB2353	*ift-20(ok3191)I*	*ift-20(null)*	OMRF Knockout Project/ CGC
GOU2362	*ift-74(cas499[ift-74::gfp])II*	IFT-74::GFP knock-in	Dr. Guangshuo Ou/CGC (Yi *et al.* 2017)
DAM456	*vieSi12[pAD373; Pccep-290::gfp::ccep-290cDNA; cb unc-119(+)]II; vieSi16[pAD390; Phyls-1::mCherry::hyls-1; cb unc-119(+)]IV*	GFP::CCEP-290 (*Mos* transposase-mediated single-copy insertion (MosSCI)) + mCherry::HYLS-1 (MosSCI)	Dr. Alexander Dammermann (Schouteden *et al.*2015)
AND16	*ift-20(ok3191)I*	*ift-20(null)* outcrossed 6x	This study, made from RB2353
AND56	*him-8(e1489)IV*	*him-8* mutant for matting assay	This study, made from OE3002
AND67	*ift-20(ok3191)I; him-8(e1489)IV*	*ift-20(null) + him-8* mutant for matting assay	This study
AND185	*ift-20(ok3191)I; ift-74(cas499[ift-74::gfp])II*	*ift-20(null) +* IFT-74::GFP	This study
AND196	*ift-20(ok3191)I; vieSi12[pAD373; Pccep-290::gfp::ccep-290cDNA; cb unc-119(+)]II; vieSi16[pAD390; Phyls-1::mCherry::hyls-1; cb unc-119(+)]IV*	*ift-20(null) +* GFP::CCEP-290 (MosSCI) + mCherry::HYLS-1 (MosSCI)	This study
AND226	*vieSi16[pAD390; Phyls-1::mCherry::hyls-1; cb unc-119(+)]IV; lqIs2[Posm-6::GFP; lin-15(+)]X*	mCherry::HYLS-1 (MosSCI) + *Posm-6::GFP*	This study, made from LE309
AND227	*ift-20(ok3191)I; vieSi16[pAD390; Phyls-1::mCherry::hyls-1; cb unc-119(+)]IV; lqIs2[Posm-6::GFP; lin-15(+)]X*	*ift-20(null) +* mCherry::HYLS-1 (MosSCI) + *Posm-6::GFP*	This study, made from LE309
